# Predicting carob tree physiological parameters under different irrigation systems using Random Forest and Planet satellite images

**DOI:** 10.3389/fpls.2024.1302435

**Published:** 2024-03-19

**Authors:** Simone Pietro Garofalo, Vincenzo Giannico, Beatriz Lorente, Antonio José García García, Gaetano Alessandro Vivaldi, Afwa Thameur, Francisco Pedrero Salcedo

**Affiliations:** ^1^ Department of Soil, Plant and Food Sciences, University of Bari “Aldo Moro”, Bari, Italy; ^2^ Department of Irrigation, Centro de Edafología y Biología Aplicada del Segura – Consejo Superior de Investigaciones Científicas (CEBAS-CSIC), Murcia, Spain; ^3^ Laboratory of Biodiversity, Molecules, Application (BMA), Higher Institute of Applied Biology Medenine, University of Gabes, Medenine, Tunisia

**Keywords:** remote sensing, physiology modeling, carob tree, machine learning, Random Forest regression

## Abstract

**Introduction:**

In the context of climate change, monitoring the spatial and temporal variability of plant physiological parameters has become increasingly important. Remote spectral imaging and GIS software have shown effectiveness in mapping field variability. Additionally, the application of machine learning techniques, essential for processing large data volumes, has seen a significant rise in agricultural applications. This research was focused on carob tree, a drought-resistant tree crop spread through the Mediterranean basin. The study aimed to develop robust models to predict the net assimilation and stomatal conductance of carob trees and to use these models to analyze seasonal variability and the impact of different irrigation systems.

**Methods:**

Planet satellite images were acquired on the day of field data measurement. The reflectance values of Planet spectral bands were used as predictors to develop the models. The study employed the Random Forest modeling approach, and its performances were compared with that of traditional multiple linear regression.

**Results and discussion:**

The findings reveal that Random Forest, utilizing Planet spectral bands as predictors, achieved high accuracy in predicting net assimilation (R² = 0.81) and stomatal conductance (R² = 0.70), with the yellow and red spectral regions being particularly influential. Furthermore, the research indicates no significant difference in intrinsic water use efficiency between the various irrigation systems and rainfed conditions. This work highlighted the potential of combining satellite remote sensing and machine learning in precision agriculture, with the goal of the efficient monitoring of physiological parameters.

## 1 Introduction

Despite the growing scarcity of water and the deterioration of its quality, irrigated agriculture plays a fundamental role in meeting the current and future demand for food production (UN-Water, 2021) which aligns with the sustainable development goal (SDG6): zero hunger set by the United Nations. Carob tree demonstrates to have at least three competitive advantages over other silviculture species: i) ranks first as an agriculture product, with very high annual revenues; ii) very resistant to drought iii) and very resistant to forest fires, in particular when compared to pine forests (Dimitrakopoulos and Papaioannou, 2001). Another added value of the carob tree is the health properties. The natural antioxidants present in plant-derived foods and products include vitamins (e.g., vitamin C) and bioactive phenolics (e.g., flavonoids, procyanidins, etc.). These natural antioxidants once ingested and metabolized can reach target cells and organs to exert a beneficial effect on health. Also, the carob tree is a rich source of phenolics and (poly)phenolics (Goulas et al., 2016). Although it may seem that carob tree has a low carbon fixation potential when compared to other species, both due to a slow growth rate and small densities, this crop has the potential to grow faster and with high density if conservative irrigation is applied and cultural practices are implemented. Therefore, Carob tree can be considered as a CO_2_ sink under the Kyoto Protocols Article 3.3, due to the lower risk of lost revenues in the future, which has been keeping investors away from the market (more on this may be found in Hamilton et al., 2010 and Chenost et al., 2010). Nowadays, in the context of sustainable agriculture, the importance of rationalizing water uses and improving its use efficiency is increasing (Garofalo et al., 2023a). To apply the correct amount of water in quantity and quality under the conservative agriculture recommendations, plant-based sensing methods provide the most precise measure of plant water status for irrigation management and water stress monitoring, as they provide the integrated response of the plant to soil moisture availability and atmospheric influences. These data could be used to train models for Decision Support System (DSS) and obtain an optimized irrigation management. Most of these variables could be continuously monitored using sensors and could provide a reliable estimate of crop water status (Fernández, 2017), but their local application does not allow the variability existing within the plot to be known. Mapping the plot heterogeneity and variability in plant and soil water status with geospatial models (Panda et al., 2010; Tsoulias et al., 2019) could support the application of precision agriculture approaches aimed at identifying and irrigating different sectors of the plot (management zones) according to their current water status. This is of much interest especially because of the huge variation of leaf water parameters (Boutasknit et al., 2020, 2021) under drought conditions and throughout the course of the growth cycle. To this, using remote sensing tools, those variations could be captured/sensed and then translated into efficient irrigation management.

### 1.2 Remote sensing in agriculture

Remote sensing consists of detecting and monitoring the properties of an object present on the surface of the Earth by measuring its reflected (or emitted) radiation at a distance (Weiss et al., 2020); these data are typically acquired by using satellites, aircraft, and more recently Unmanned Aerial Vehicles (UAVs), with accurate results and affordable costs (Corwin and Scudiero, 2019). The application of remote sensing technologies in agriculture has greatly increased in recent past years (Weiss et al., 2020). UAVs images have been successfully employed to predict several crop traits, for example, Kasper et al. (2019a and 2019b) used UAV imagery to predict the salt-stressed tomato biomass and yield at harvesting time and to map the phenotypic traits, providing farmers with a way to monitor the effects of salt stress during the plant cycle. Nevertheless, the use of aircraft and drones is still expensive and requires operation time; using satellite images to estimate plant-related parameters could be a viable option to obtain spectral images to process for agricultural issues with a low cost and relatively higher rapidity. Also, the application of GIS software (*Geographic information system*, e.g., QGIS) is proving to be useful for mapping the variability of several parameters existing at field and regional scale (Alhajj Ali et al., 2023). In agricultural applications, three different approaches are commonly used with remote sensing data to map the variability of the agronomic or biophysical plant traits within the field: parametric, physical-based, and non-parametric. Parametric approaches are the easiest to study the relationship between remote sensing data and crop variables, nonetheless, they require that specific assumptions be respected (e.g., normal distribution of the data); physical-based approaches (e.g., radiative transfer models) are based on physical laws, but due to their complexity the applicability is low; non-parametric approaches (e.g., machine learning methods) are useful for their abilities to find hidden information and relationship between the data and low sensitivity to non-normally distributed data (Jiang et al., 2022). The use of machine learning, combined with remote sensing data, has greatly grown in recent years in many areas of agriculture (Sharma et al., 2021a). One of the most widely used machine learning algorithms in agricultural remote sensing applications is the supervised ensemble-learning algorithm random forest, for solving classification and regression problems (Belgiu and Drăgu, 2016). Random forest has been used in different remote sensing applications; Jiang et al. (2022) demonstrated that the combination of UAV-based remote sensing and machine learning can predict important traits of quinoa under abiotic stress conditions. In addition, machine learning has also been used in combination with satellite remote sensing. For example, Laroche-Pinel et al. (2021) used Sentinel-2 imagery to map vine water status in the south of France, comparing the performances of different machine learning algorithms.

The aim of the presented research work was the developing of machine learning models to predict net assimilation and stomatal conductance of the carob tree, using satellite bands reflectance data as predictors; additionally, these models were used to understand the differences in the aforementioned parameters across the various irrigation systems.

## 2 Materials and methods

### 2.1 Experimental farm conditions

**Figure 1:**
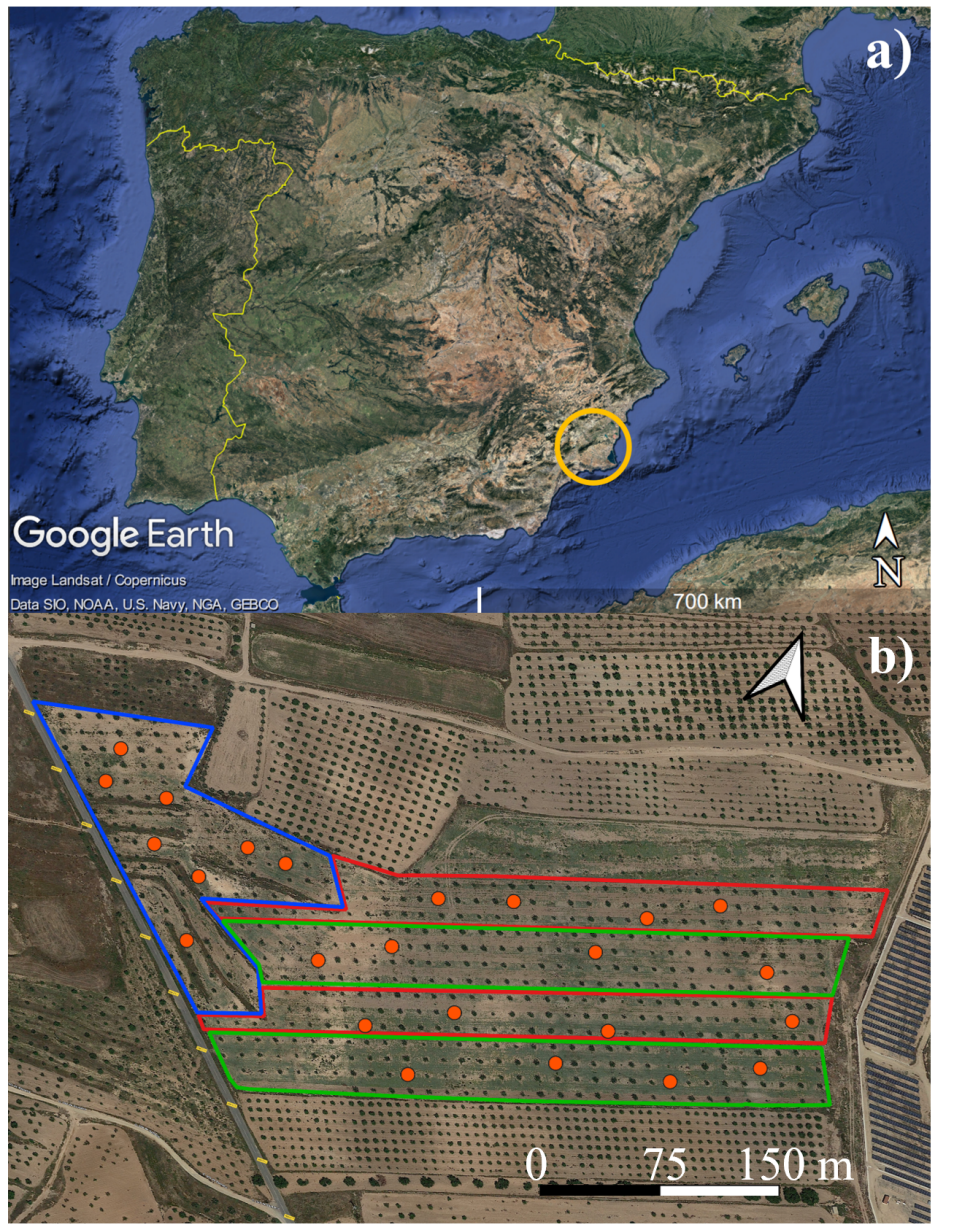
**(A)** Murcia region, Spain (yellow ring); **(B)** experimental field, red areas indicate zones under subsurface drip irrigation system with 2.3 liters per hour (SDI 2.3 L) green areas indicate zones under subsurface drip irrigation system with 1.6 liter per hour (SDI 1.6 L), white area indicate rainfed zone (RD). Orange circles indicate carob trees used to acquire ground data. Images from Google Earth Pro for desktop (7.3.6.9345); Map data ^©^2015 and ^©^2020, Google.

The experiment was conducted in 2023, at an 8-ha commercial carob tree orchard (*Ceratonia siliqua*, L., 1753; cv. *Ramillete*) located in Fuente Álamo de Murcia, Murcia region (South-East of Spain; 37°45’57.1”N, 1°14’39.6”W; 218 m a.s.l.) (**Figure 1**); the orchard was planted in 2014 with a spacing of 12 x 12 m between trees and rows. The soil has been characterized at two distinct depths, 30 cm and 90 cm, with 10 soil samples collected at representative points across the farm for each depth. These samples have been subsequently mixed based on their respective depths. Soil parameters were analyzed by a private laboratory in Murcia (Fitosoil Laboratories, Alcalde Clemente García, 24/37, 30169 San Ginés – Murcia). The soil texture within the first 30 cm depth was classified as clay loam (36% clay, 38% silt, and 26% sand), with an average bulk density of 1.37 g cm^−3^, a pH of 7.67 and electrical conductivity of 180 µS/cm. Within the first 90 cm depth it had a loamy texture (22% clay, 30% silt, and 48% sand) with an average bulk density of 1.49 g cm^−3^), a pH of 7.85 and electrical conductivity of 154 µS/cm. In **Supplementary Materials**, the complete results of the soil analyses at the two reported depths are presented. The orchard management followed the organic farming guidelines, fertilizers were not applied during 2023. Carob trees were harvested on the 9th of August; 2023 was not a productive year, in fact, the average production per tree was 14 kg.

The climate of the experimental area is Mediterranean, classified as BSk, according to Köppen and Geiger; the amount of annual rainfall is generally little (321 mm), the driest month is July (3 mm), the warmest month is August (average temperature of 25.5°C) and the coldest is January (average temperature of 10.3°C) (Climate-Data.org, 2023). Data related to reference evapotranspiration (ETo), average temperature, rainfall, and vapor pressure deficit (VPD) during the period of the experiment were provided by the agricultural information system of Murcia (“*Sistema de información agrario de Murcia*”).

The irrigation system was installed 7 years after the orchard planting, and it consisted of a double lateral drip line laid on the subsurface soil at 0.30 cm 1.5 m from the tree trunk and 1.5 between the drip lines. It provided three self-pressure compensating on-line emitters per tree discharging 2.3 L h^-1^ and 1.6 L h^-1^, spaced 1 m apart. The irrigation water quality was a mix of rainwater harvesting and well water with an EC of 1.76 dS/m and a pH: 7.87.

The orchard was divided into three zones under different irrigation management: subsurface drip irrigation with 2.3 liters per hour (SDI 2.3 L), subsurface drip irrigation with 1.6 liters per hour (SDI 1.6 L) and Rainfed (RD) (**Figure 1**). Irrigation was applied eight times during the year on the critical phenological stages for carob tree (Correia and Martins-Loução, 2004; Tous et al., 2013) - emergency of axillary and apical buds, floral induction, fruit growth, post-harvesting - on DOYs 10, 114, 135, 136, 198, 233, 240 and 247, for a total amount of 322.56 m^3^/ha (SDI 1.6 L) and 463.68 m^3^/ha (SDI 2.3 L); considering that, as for other rainfed species there are no literature reference for irrigating carob trees, it was decided to apply the same irrigation time for all the irrigation interventions, except for the first two (12 hours of irrigation instead of 6, as for the following ones). **Table 1** reports the amount of water applied per each irrigation intervention.

**Table 1:** Amount of water applied per irrigation intervention in the year of the experiment for both the irrigation systems: subsurface drip irrigation with 1.6 liter per hour (SDI 1.6 L) and subsurface drip irrigation with 2.3 L per hour (SDI 2.3 L).

DOY	m^3^/ha	
	SDI 1.6 L	SDI 2.3 L
10	64.53	92.76
114	64.53	92.76
135	32.25	46.36
136	32.25	46.36
198	32.25	46.36
233	32.25	46.36
240	32.25	46.36
247	32.25	46.36
**Total**	322.56	463.68

### 2.2 Plant physiological measurements

A gas exchange system (LI-6400, LI-COR Inc., Lincoln, NE, USA) was used to determine net assimilation – the amount of carbon dioxide used by the leaves per square meter per second - (Pn, µmol CO2 * m-2 s-1) and stomatal conductance – the stomatal gas exchanges per square meter per second - (gs, mmol H2O * m^-2^ s^-1^); these parameters were acquired between 11.00 to 13.00 hr solar at light saturation (PAR ≥ 1600 mol photons * m^-2^ s^-1^) on healthy, mature, fully expanded and sun-exposed leaves on 8 trees per irrigation system. The intrinsic water use efficiency (iWUE) was calculated as the ratio between Pn and gs (Haider et al., 2018). Measurements were taken around irrigation days and during the growing season up to pre-harvest.

### 2.3 Satellite images

Planet Labs PBC (from now on “Planet”) is an American company that is specialized in capturing high-resolution spectral images of Earth with high frequency. The spectral images were acquired from the third-generation satellite of PlanetScope (Equator crossing time of 7.30-11.30 a.m., local solar time) with eight spectral bands (PBs) and a spatial resolution of 3 meters (Imagery^©^ 2023, Planet Labs PBC, San Francisco, CA, USA); the PBs were coastal blue (431-452 nm), blue (465-515 nm), green I (513-549 nm), green (547-583 nm), yellow (600-620 nm), red (650-680 nm), red edge (697-713 nm) and NIR (845-885 nm) (Planet Imagery Product Specifications, 2022). All the images were downloaded from the online tool “Planet explorer” (www.planet.com/explorer) as orthorectified and radiometrically corrected TIFFs to maintain consistency across localized atmospheric conditions and to reduce the uncertainty of the spectral response in time and place to a minimum (Planet Imagery Product Specifications, 2022). Then the images were converted to surface reflectance following the instructions in the metadata provided by Planet. For each tree considered in the study, the reflectance was considered as the mean value of the reflectance of the pixels by using the plugin “Zonal statistics” in QGis (Qgis, 2023).

### 2.4 Statistical analysis and machine learning

In this work, Pn and gs measured on field were independently considered as response variables, using the reflectance of the 8 PBs as predictors; each dataset (n = 217) was randomly split into a training dataset (80%) and a testing dataset (20%). The training dataset was used to fit the developed model and the testing dataset was used to test the model performance and robustness. The machine learning algorithm random forest (RF) was used to predict the variables. RF is a supervised ensemble-learning algorithm that improves regression combining multiple decision trees to enhance the accuracy of the model and its generalization. Due to its accuracy and ability in finding non-parametric relationships, RF is used in the fields of remote sensing and agronomy for prediction and modeling (Belgiu and Drăgu, 2016; Nayak et al., 2022; Silva et al., 2023). In this work, RF model implemented in the “ranger” package in RStudio was used (Wright and Ziegler, 2017); to avoid the overfitting of the model, the *10-fold-cross-validation* was applied, by using the *trainControl* function of the package “caret” (Kuhn, 2008). In the ranger implementation of RF model, several hyper-parameters were fine-tuned, including the quantity of the variables to potentially split each node (mtry), the splitting rule, and the minimum size of the node. The fine-tuning model procedure involved several iterations of these parameters, except for the number of trees, fixed at 500, due to the absence of a significant impact on the global performance of the model. The importance of each variable was assessed through permutation (Janitza et al., 2013). Furthermore, a linear model (LM) for each variable to predict was trained with the goal of establishing a baseline useful for the evaluation of the results; in LM, avoiding using all the predictors, only the statistically significant variables were maintained (*p*< 0.05). To compare the performance of the models and their robustness, coefficient of determination [R^2^ (Equation 1)], root mean square error [RMSE (Equation 2)], normalized root mean square error [nRMSE (Equation 3)] and mean absolute error [MAE (Equation 4)] were calculated as follows:


(1) $$R^{2}=1-\frac{\Sigma_{\text{i}=1}^{n}{\left(E_{i}-S_{i}\right)}^{2}}{\Sigma_{\text{i}=}^{n}{\left(E_{i}-\underline{E_{i}}\right)}^{2}}$$

(2) $$RMSE=\sqrt{\frac{1}{n}\sum_{i=1}^{n}{\left(S_{i}-E_{i}\right)}^{2}}$$

(3) $$nRMSE=100\frac{\sqrt{\frac{1}{n}\sum_{i=1}^{0}{\left(S_{i}-E_{i}\right)}^{2}}}{nval}$$

(4) $$MAE=\frac{\sum_{i=1}^{n}\left|S_{i}-E_{i}\right|}{n}$$

where *S* were the simulated values, *E* the expected values and *n* the number of the observations.

Finally, by using the package “raster” (Hijmans and van Etten, 2012), the RF-based model was applied to further 16 spectral images downloaded from Planet Explorer to model the trend of the carob trees physiological parameters from January to September.

To detect the statistical differences between the irrigation system, the Analysis of Variance test (ANOVA) was carried out, followed by Tukey’s multiple comparisons test, with significance level set at 0.05.

RStudio software (RStudio, 2020 for Windows, Version 2023.06.0 + 421, PBC, Boston, MA) and SigmaPlot (SigmaPlot, Systat Software Inc, Version 14 for Windows) were used for machine learning and statistical analyses, modeling, and graph plotting.

## 3 Results

### 3.1 Field data

#### 3.1.1 Climatic conditions

**Figure 2:**
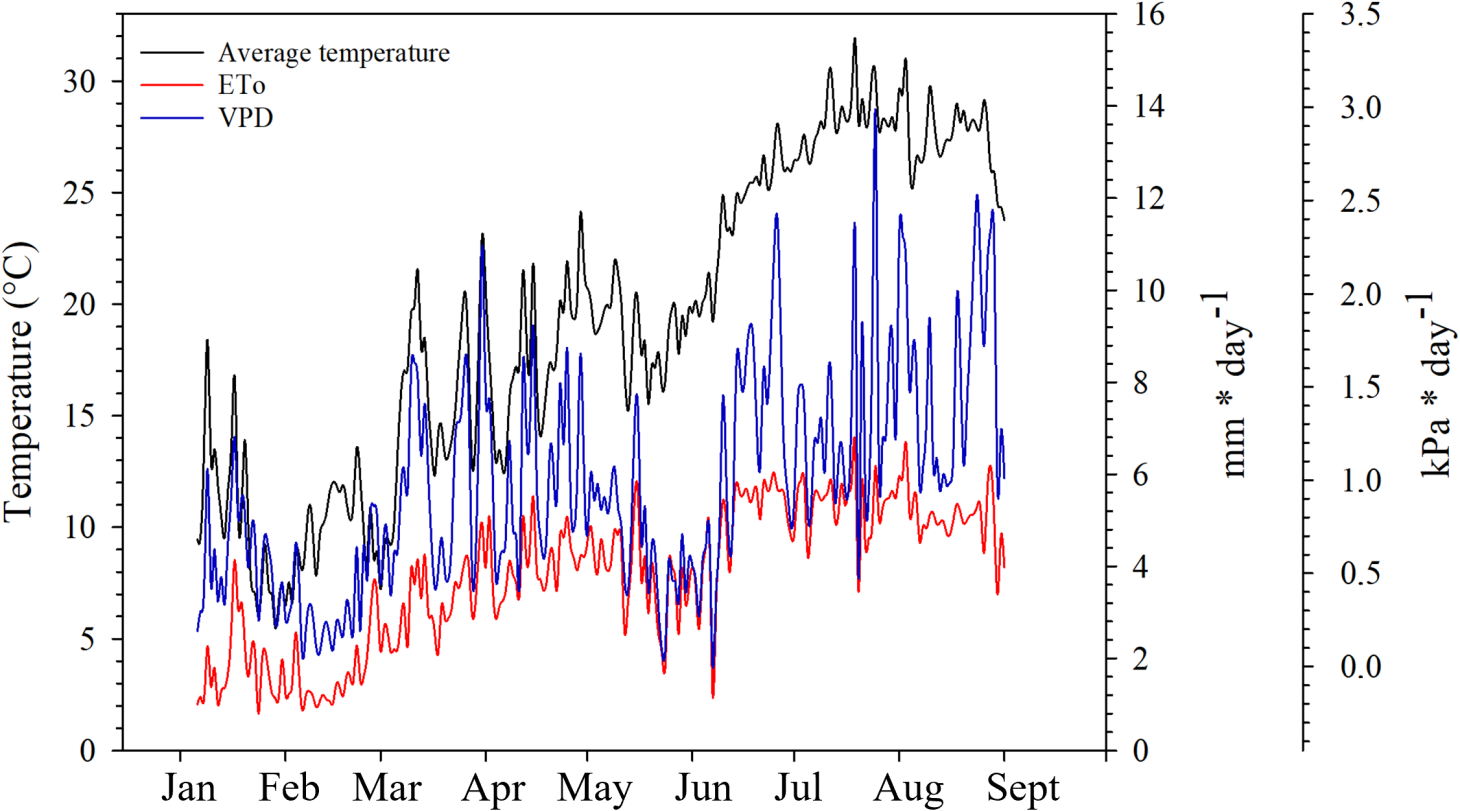
Daily variation of average temperature, reference evapotranspiration (ETo) and Vapour Pressure Deficit (VPD) in the area of the experiment (Fuente Álamo de Murcia, Spain).

During the experiment, the lowest average temperature was recorded in January on DOY 29 (5.4°C) and the highest in July on DOY 200 (31.89°C); during the growing season, the daily average temperature remained around 20°C in the first part of May, until DOY 131, reaching 24.14°C on DOY 119, then dropped to temperatures around 16°C and started to increase again in the last part of May and June; particularly, during the summer and until the harvest the daily average temperature remained above 25.24°C; the highest monthly values of ETo there were in June (154.46 mm) and in July (172.02 mm). The lowest values of VPD were recorded in the second half of May and June, peaks of VPD have occurred in June (DOY 177, 2.42 kPa), July (DOY 200, 2.33 kPa and DOY 206, 2.98 kPa) and August (DOY 214, 2.31 kPa) (**Figure 2**). The rainiest months were May (84.10 mm concentrated in the last 10 days of May) and June (14.50 mm concentrated in the first 10 days of June), the driest months were July (2.80 mm) and August (0.20 mm).

#### 3.1.2 Plant physiological parameters

**Figure 3:**
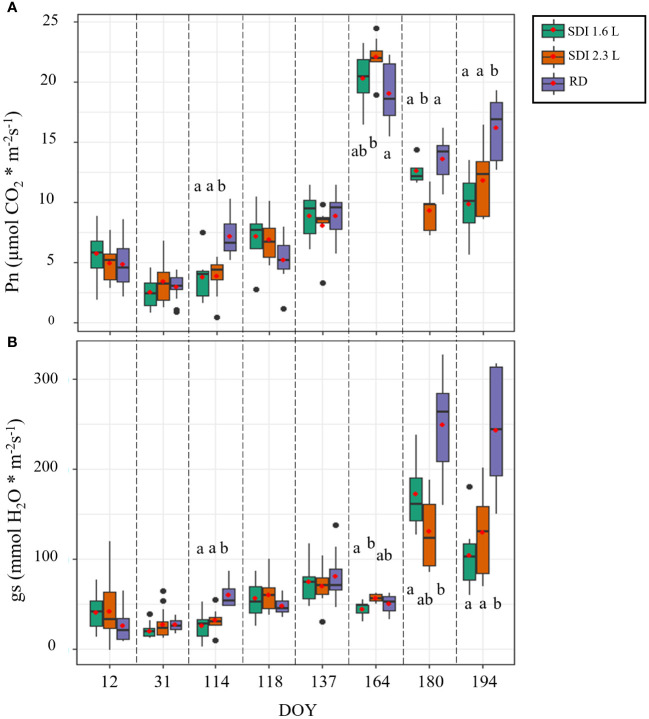
Boxplot of the field data used to train the models; **(A)** net assimilation (Pn) and **(B)** stomatal conductance (gs) of the carob trees under subsurface drip irrigation with 1.6 l/h (SDI 1.6 L), subsurface drip irrigation with 2.3 l/h (SDI 2.3 L) and rainfed carob trees (RD). Letters indicate significant differences among the systems (p< 0.05).

From January to May, until DOY 137, the level of Pn was generally low: over this period, the means of the Pn values of both SDI systems and RD remained below 10 µmol CO_2_ * m^-2^ s^-1^. In DOY 118, after the second irrigation application, no statistically significant difference was observed between the SDI systems and RD, but an increase of Pn was recorded for both SDI systems compared with the previous date, except for RD. A peak of the values was registered in the middle of June (DOY 164) for both systems and RD; then, in the last two measurement dates (DOY 180 and 194) the values of Pn dropped again, those of RD remained higher than the two irrigation systems, in Doy 180 SDI 2.3 L carob trees showed significant lower Pn (12.06 µmol CO_2_ * m^-2^ s^-1^) than SDI 1.6 L and RD carob trees; in DOY 194 Pn of RD carob trees was significantly higher than SDI 2.3 L and SDI 1.6 L carob trees (**Figure 3A**).

For most of the measurement period, gs remained stable and low until June (average gs values lower than 90 mmol H_2_O * m^-2^ s^-1^), with a slight increase on the measurement date after the second irrigation (DOY 118), when the medians of both the irrigation systems were higher than those recorded on the previous date; on DOY 114 RD, gs was significantly higher (59.94 mmol H_2_O * m^-2^ s^-1^) than SDI 2.3 L and SDI 1.6 L, on DOY 118 no statistical significant differences were found between the irrigation systems and RD. On DOY 164, SDI 2.3 L carob trees showed significantly higher values of gs (56.68 mmol H_2_O * m^-2^ s^-1^) compared with SDI 1.6 L, but not compared with RD carob trees. Generally, high values of gs were recorded in the two last dates of measurement (DOY 180 and 194), with higher values for RD in both dates (**Figure 3B**).

### 3.2 Random forest and linear model prediction performance

#### 3.2.1 Random forest

**Figure 4:**
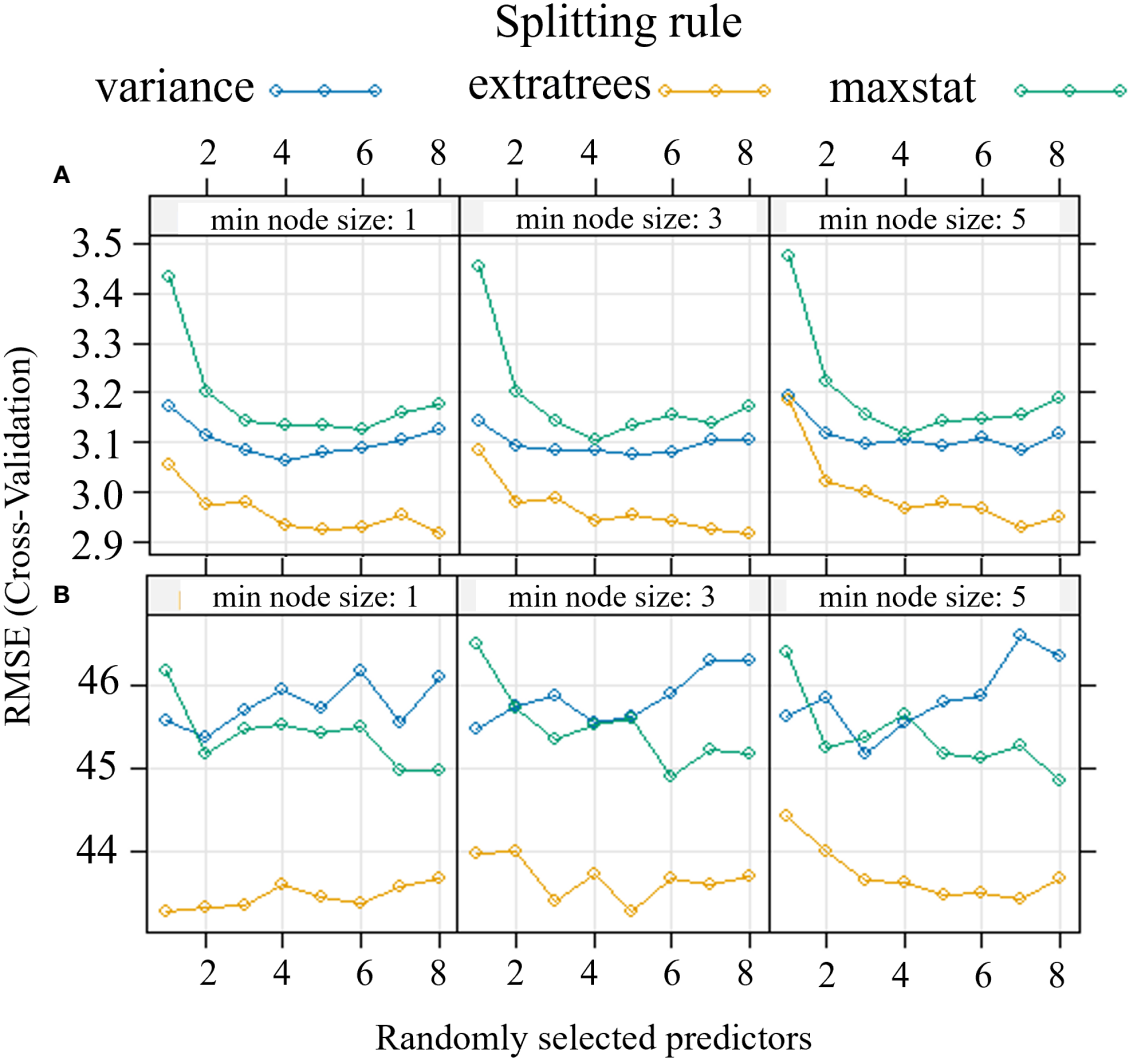
Optimization of Random Forest parameters (splitting rule; min. node size; mtry) for the predictive model of net assimilation **(A)** and stomatal conductance **(B)** of the carob tree under different drip irrigation systems.

**Figure 5:**
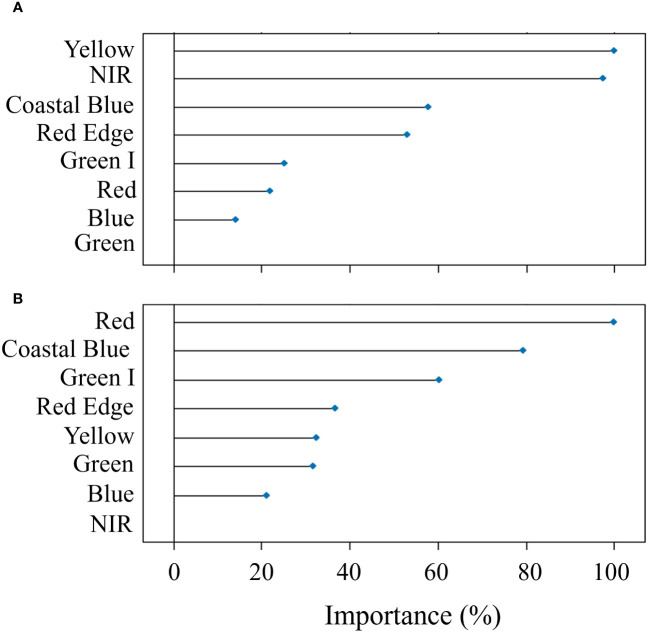
Results of the permutation process showing the importance (%) of the predictors in Random Forest modeling used for the prediction of net assimilation **(A)** and stomatal conductance **(B)** of the carob tree under different drip irrigation systems.

The modeling procedure to predict Pn involving RF in training had good fit (R^2^ = 0.96) and low error (nRMSE = 4.6%), the performance of the model in testing was positive, with an R^2^ = 0.81 and a nRMSE of 11.7% (**Table 2**). The results of the optimization of the model parameters are reported in **Figure 4A**: based on the lowest RMSE value, 8 variables as mtry, extratrees as splitting rule and 1 as minimum node size were used in the final model. The two most important bands in the prediction of Pn were yellow and NIR (**Figure 5A**).

**Table 2:** Performance parameters of Random Forest (RF) and Linear Model (LM) predicting stomatal conductance (gs) and net assimilation (Pn).

	Training					Testing			
		R^2^	RMSE	nRMSE	MAE	R^2^	RMSE	nRMSE	MAE
**Pn**	**RF**	0.96	1.09	4.6	0.84	0.81	2.31	11.7	1.79
	**LM**	0.53	3.90	16.3	3.08	0.52	3.71	18.7	2.90
**gs**	**RF**	0.89	20.89	6.7	12.92	0.70	36.84	11.6	25.44
	**LM**	0.46	46.17	14.5	34.06	0.35	56.14	17.9	39.32

In the prediction of gs, RF had a R^2^ = 0.89 and a nRMSE of 6.7% in training and maintained good performance in testing (R^2^ = 0.70 and nRMSE = 11.6%) (**Table 2**). **Figure 4B** shows the results of the optimization of the model parameters: based on the lowest RMSE value, 5 variables as mtry, extratrees as splitting rule and 3 as minimum node size were used in the final model. The two most important bands in the prediction of gs were red and coastal blue (**Figure 5B**).

#### 3.2.2 Linear model

In the prediction of Pn, LM showed low fit in training with a R^2^ = 0.53 and nRMSE = 16.3% and poor performance in testing (R^2^ = 0.52; nRMSE = 18.7%) (**Table 2**). Based on the *p*-value, the predictors maintained in LM were the spectral bands: coastal blue, green, yellow, red, red edge and NIR.

In the prediction of gs, results were similar in terms of performance parameters (R^2^ = 0.35 and nRMSE = 17.9% in testing) (**Table 2**). The spectral bands maintained in LM for the prediction of gs were blue, green I, yellow and red edge.

### 3.3 Remote sensing physiology modeling

Each RF-based model found for the prediction of the physiological parameters was applied to further Planet spectral images; thus, information on physiological patterns of the carob trees were obtained even in the phase of the growing season in which there were no field data.

#### 3.3.1 Predicted net assimilation

**Figure 6:**
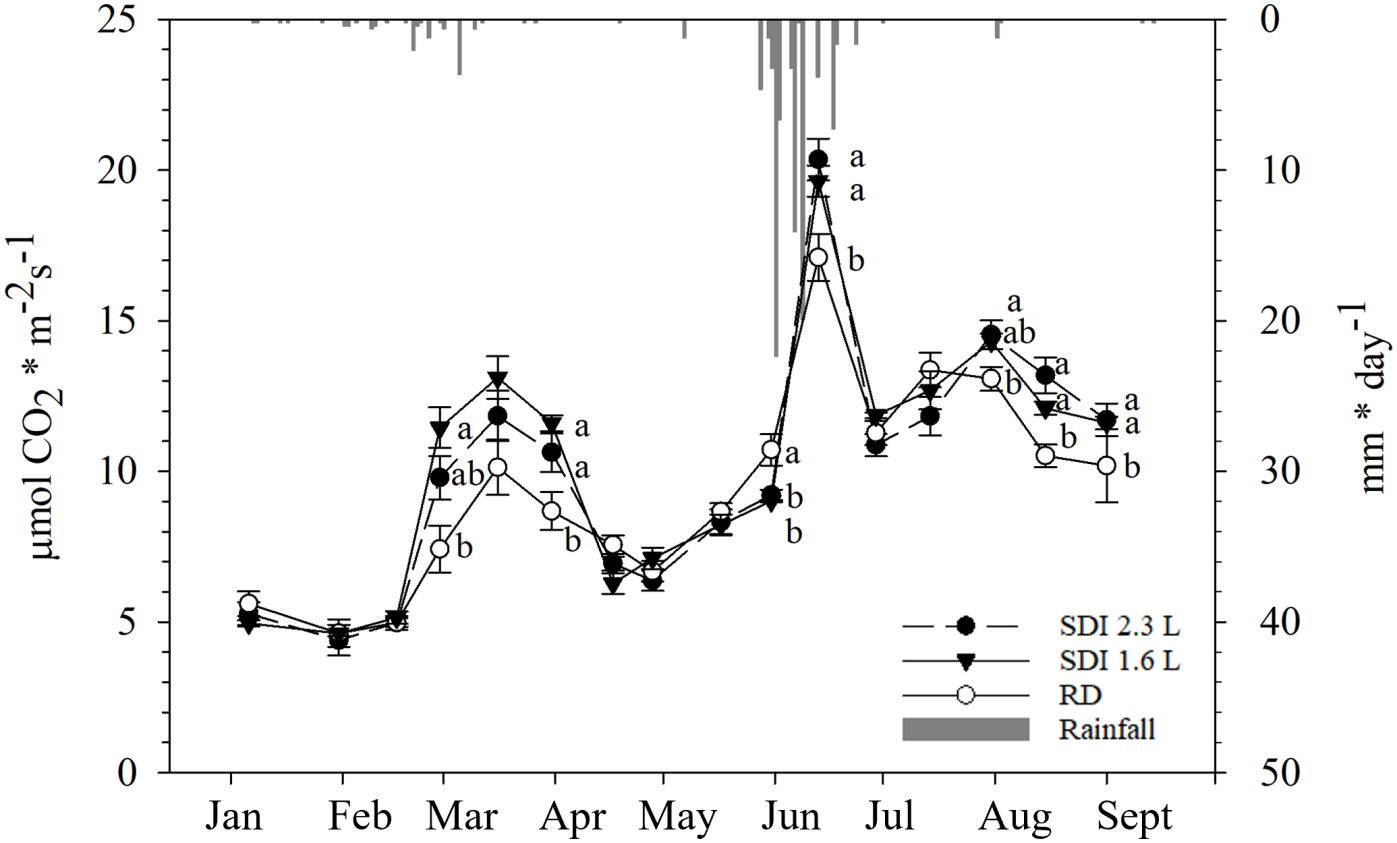
Inter-seasonal trend (mean and standard error) of net assimilation (Pn) of subsurface drip irrigated with 2.3 l/h (SDI 2.3 L) and 1.6 l/h (SDI 1.6) carob trees and rainfed (RD) carob trees, predicted with Random Forest. Letters indicate significant differences among the systems (p< 0.05).

The values of Pn remained low until mid-February, without differences among the systems and RD. From the end of February values started to increase until mid-March, particularly, at the end of February SDI 1.6 L Pn (11.45 µmol CO_2_ * m^-2^ s^-1^) was significantly higher than RD (7.42 µmol CO_2_ * m^-2^ s^-1^), but not statistically significantly different than SDI 2.3L (9.79 µmol CO_2_ * m^-2^ s^-1^). From the end of March to the end of April, Pn decreased and no statistically significant differences there were among the systems and RD; at the end of March Pn values of SDI 2.3 L and SDI 1.6 L (11.84 and 13.11 µmol CO_2_ * m^-2^ s^-1^, respectively) were significantly higher than RD (10.31 µmol CO_2_ * m^-2^ s^-1^). After the third irrigation in mid-May, no statistically significant differences were found between the systems and RD, then Pn values started to increase in all the systems, reaching a peak in mid-June, when Pn of SDI 2.3 L and SDI 1.6 L carob trees (20.35 and 19.63 µmol CO_2_ * m^-2^ s^-1^, respectively) were statistically significant higher than RD carob trees (17.10 µmol CO_2_ * m^-2^ s^-1^); at the end of June Pn values dropped again. From mid-July a weak increasing recovery of Pn was recorded for all the systems, with a further decline in mid-August, when SDI 2.3 L and SDI 1.6 L Pn (13.19 and 12.09 µmol CO_2_ * m^-2^ s^-1^, respectively) were significantly higher than RD Pn (10.51 µmol CO_2_ * m^-2^ s^-1^) (**Figure 6**).

#### 3.3.2 Predicted stomatal conductance

**Figure 7:**
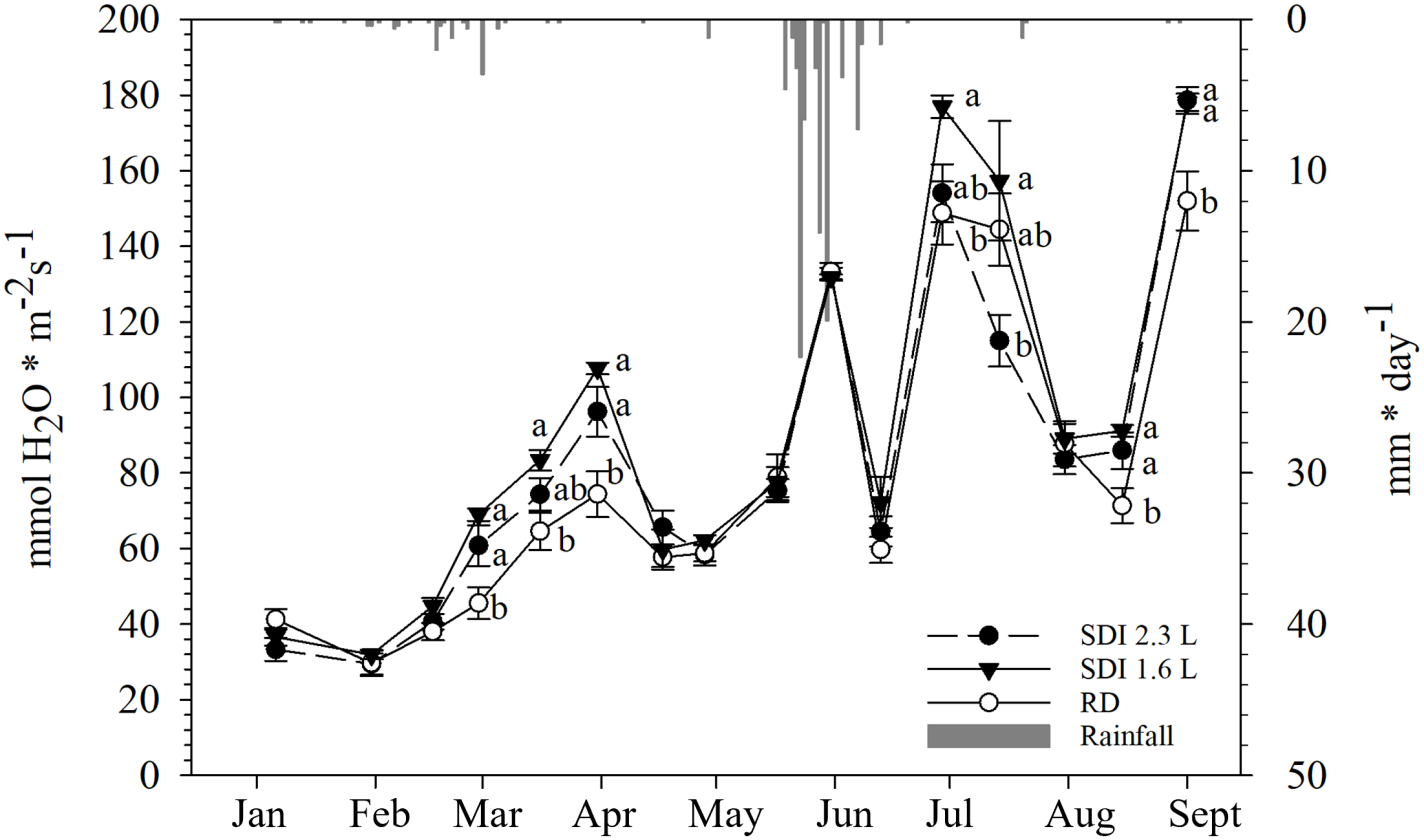
Inter-seasonal trend (mean and standard error) of stomatal conductance (gs) of subsurface drip irrigated with 2.3 l/h (SDI 2.3 L) and 1.6 l/h (SDI 1.6) carob trees and rainfed (RD) carob trees, predicted with Random Forest. Letters indicate significant differences among the systems (p< 0.05).

From January to mid-February no statistically significant differences were observed between the systems and RD; from the end of February gs increased until the end of March, with significantly higher values observed on carob trees under SDI 2.3 L and SDI 1.6 L systems (60.78 mmol H_2_O * m^-2^ s^-1^ and 68.99 mmol H_2_O * m^-2^ s^-1^, respectively) than RD (45.52 mmol H_2_O * m^-2^ s^-1^). Then, from mid-April, gs decreased in all systems and RD, and no statistically significant differences were found after the second and the third irrigation application. At the end of March gs values of both systems and RD markedly increased and then sharply decreased again in mid-June, without any significant difference. At the end of June a peak of gs values, with significantly higher gs for SDI 1.6 L carob trees (176.93 mmol H_2_O * m^-2^ s^-1^) than RD carob trees (148.81 mmol H_2_O * m^-2^ s^-1^), but not significantly differences were observed between SDI 1.6 L and SDI 2.3L and between SDI 1.6 L and RD. Afterwards, gs values dropped again for all systems and RD until mid-August, when gs was significantly higher for irrigated carob trees than RD carob trees (**Figure 7**).

#### 3.3.3 Intrinsic water use efficiency

**Figure 8:**
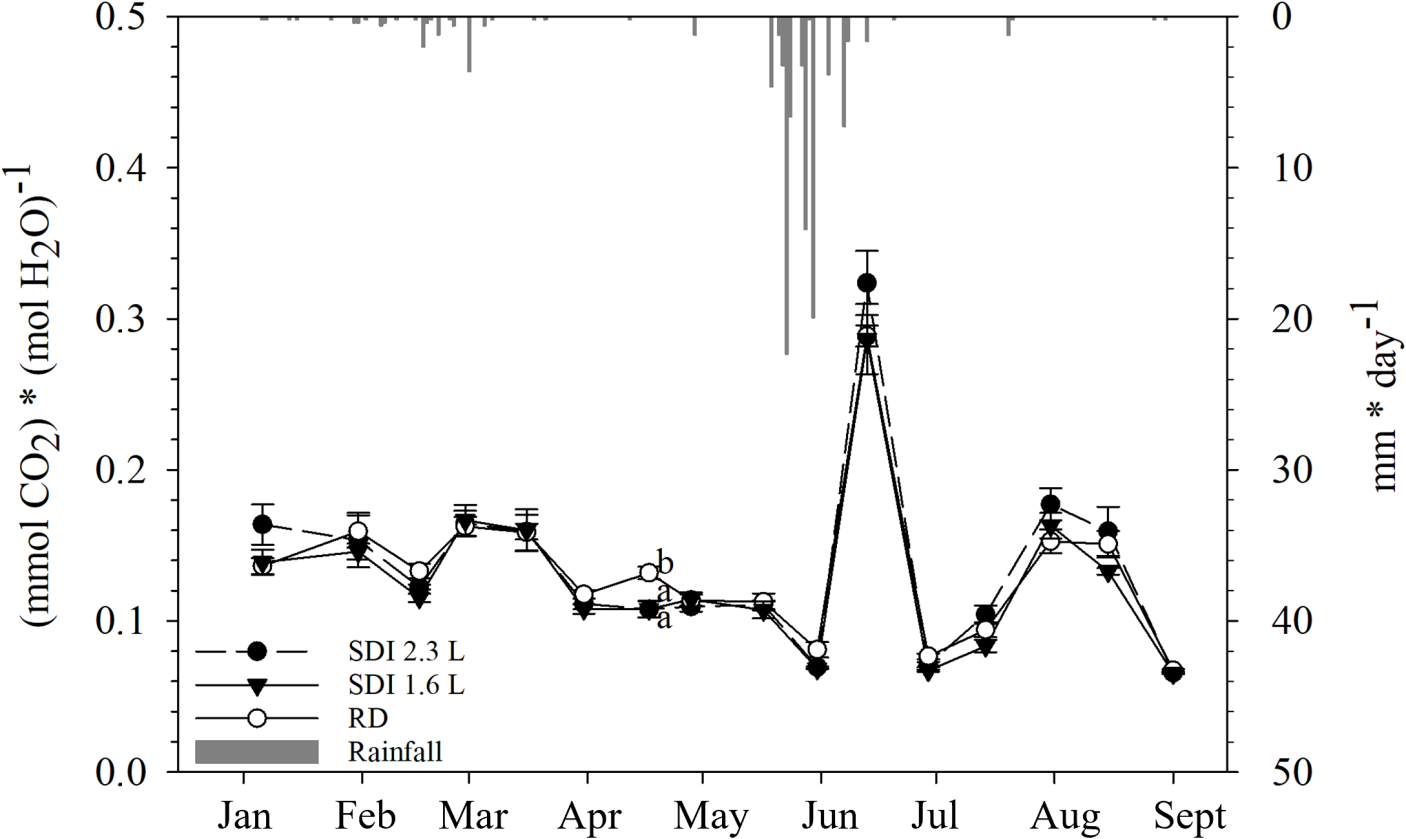
Inter-seasonal trend (mean and standard error) of intrinsic water use efficiency (iWUE) of subsurface drip irrigated with 2.3 l/h (SDI 2.3 L) and 1.6 l/h (SDI 1.6) and rainfed (RD) carob trees, predicted with Random Forest. Letters indicate significant differences among the systems (p< 0.05).

Calculated as the ratio between predicted-Pn and predicted-gs, iWUE had no statistically significant differences among the systems until mid-April, when the RD iWUE was significantly higher than the two irrigation systems; after the second irrigation application the SDI 2.3 and SDI 1.6 iWUE remained stable and RD iWUE decreased, without statistically significant differences; after the third irrigation, iWUE values were comparable with the previous and dropped at the end of May. Higher values of iWUE were obtained in mid-June for both systems and RD, without significant differences; in mid-June iWUE dropped and started to rise gradually from mid-July (**Figure 8**).

#### 3.3.4 Relationship between the physiological parameters

**Figure 9:**
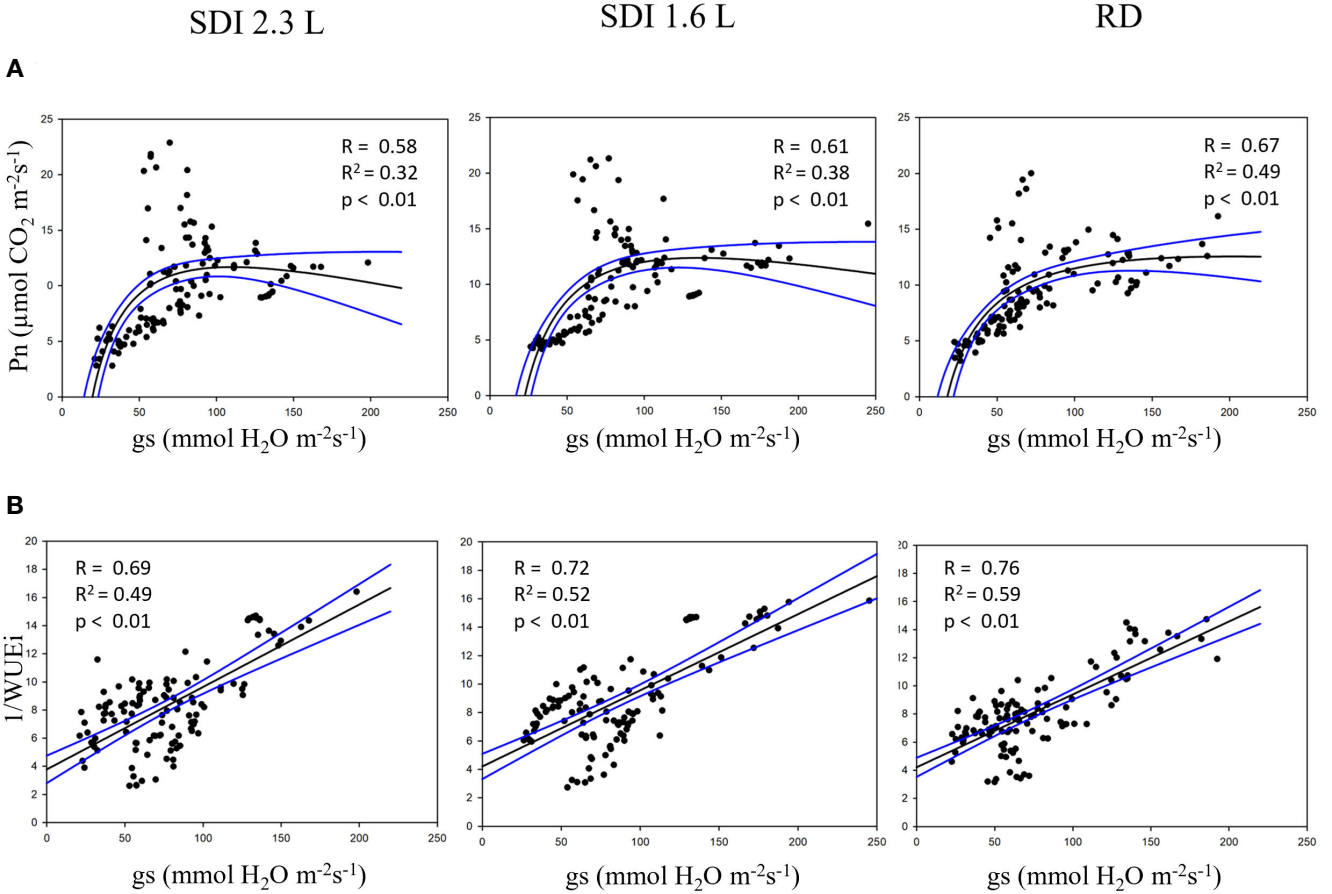
**(A)** relationship between net assimilation (Pn) and stomatal conductance and **(B)** relationships between the reciprocal of the water use efficiency (1/iWUE) and stomatal conductance of subsurface drip irrigated with 2.3 l/h (SDI 2.3 L) and 1.6 l/h (SDI 1.6) and rainfed (RD) carob trees, predicted with Random Forest.

The relationship between predicted-Pn and predicted-gs was investigated for both systems and RD, in each case the relationship was significant (*p<* 0.01); nonetheless the higher R^2^ (0.49) was found for RD carob trees, and the lower (R^2 =^ 0.32) for SDI 2.3 L carob trees (**Figure 9A**). Furthermore, the relationship between the reciprocal of the iWUE and gs was significant (*p<* 0.01) for the SDI and RD carob trees, with a higher R^2^ (0.59) for RD carob trees than SDI carob trees (**Figure 9B**).

## 4 Discussion

Crop monitoring is a key factor in understanding the response of plants to the environment and agronomic practices; nonetheless, it requires time-consuming fieldwork and efforts in order to get sufficiently representative data (Sishodia et al., 2020). Field measurements require a lot of effort to obtain data sufficiently representative from a spatial and temporal point of view, resulting in time-consuming and expensive work for farmers and technicians. In this work a method based on the integration of machine learning and remote sensing techniques has been proposed, with the purpose of having a tool to understand the variability of Pn and gs between systems and over time, reducing fieldwork.

According to the performance parameters of the RF-based modeling procedure, high accuracy in predicting both the physiological parameters considered was obtained in this work. RF has been widely employed in remote sensing applications for classification problems (Belgiu and Drăgu, 2016), while few studies have been conducted to predict continuous data. Until now, the combination of RF and remote sensing has been used more in forestry than agriculture; for example, D’este et al. (2021) implemented an RF regression algorithm using satellite data to estimate fine dead fuel and improve fire risk assessment. In agriculture, RF has mainly been used to predict the yield of herbaceous crops (Johansen et al., 2020; Dhillon et al., 2023); furthermore, Lee et al. (2020) used RF and UAV spectral imagery to predict nitrogen management in maize, achieving good results (R^2 =^ 0.85). For agricultural issues and in combination with satellite data, RF modeling has had less application, especially considering high-resolution satellite images (Planet imagery). The results obtained in the present research are comparable to those obtained by Garofalo et al. (2023b) in the prediction of the water status of the olive tree (stem water potential) in the south of Italy, using Planet imagery and an RF model; moreover, the authors found that RF outperformed the LM, as in the present research for both the targeting variables considered. Thus, these results confirm the applicability and the benefits of combining the machine learning approaches and data from high-resolution satellites. Nevertheless, it should be considered that other satellite platforms (e.g., Sentinel 1 and 2, Landsat 8) provide images freely, instead of Planet, whose images are not available for free; this could certainly represent a limitation of the applicability of the workflow presented in this study in commercial farming.

The results of the variable importance in RF modeling suggest that the spectral bands used as predictors didn’t have the same power to estimate the physiological variables. In the prediction of Pn, the yellow band had the highest importance, appearing directly linked to the photosynthetic rate of the leaves. In a previous study, Adams et al. (1999) found that a vegetation index considering the spectral region of yellow, the Yellowness Index, could measure leaf stress linked to alterations in pigment absorption, particularly chlorophyll; total chlorophyll content is well known to be closely associated with the photosynthetic rate, due to the requirement of chlorophyll molecules in driving the electron transport reaction (Buttery and Buzzell, 1977; Croft et al., 2017). In the prediction of gs, the most important band was red; according to Rapaport et al. (2015), this spectrum region is generally related to pigments that could react to water stress (e.g., xanthophyll), which directly affects gs through osmotic stress (Chaves et al., 2009), explaining the importance of the red band for the gs prediction found in this study.

Given the good performance of RF-based approach, the developed models were utilized to predict and analyze the seasonal trends of Pn and gs, and then to calculate the iWUE. The behavior of Pn and gs appeared linked to temperature, considering that, generally, with higher temperatures, Pn increases up to an optimum temperature, and gs increase exponentially (Yamori et al., 2005). Probably, the peak in Pn occurred in mid-March was due to the precipitations that fell in the first half of the month. The sharp decrease of gs found in mid-June could be explained by an asphyxia condition caused by soil flooding (as shown in the results section, a large amount of rainfall fell in May and June), in fact a reduction in oxygen concentration in the root zone could determine a rapid decline of gs (Smith et al., 1989; Barickman et al., 2019). Nevertheless, waterlogging could also affect the activity of photosynthetic enzymes, resulting in decreased Pn (Sharma et al., 2021b); during the above-mentioned period, as explained, a reduction of gs was recorded, but Pn had a peak, probably due to the optimal high temperatures recorded in the first part June and the rainfall occurred before the waterlogging; moreover, the negative effects of waterlogging might be manifested more gradually on Pn than gs (Yordanova and Popova, 2007), in fact, a sharp decline of Pn was observed at the end of June, when, on the other hand, gs had recovered, maybe due to high temperatures and no precipitations in the second part of June, resulting in evaporation of the water from the soil and then better conditions of the root system. However, based on the current knowledge it is not possible to determine with accuracy the temporal dynamics of the effects of waterlogging specifically on carob trees. iWUE also had a peak in mid-June, suggesting that under waterlogging conditions, carob tree might prevent the stress driven by soil flooding with a reduction of water losses, as also reported in a study on pepper (Ou et al., 2011); in another study on forest tree species (*Schinus terebinthifolius* and *Rapanea ferruginea*), flooding system significantly increased iWUE compared to the control (Mielke et al., 2005). August was the hottest month of the season, Pn slightly declined and gs sharply increased until September, confirming the previously mentioned relationship with the temperature trend; furthermore, irrigation was applied after the harvest, leading to higher values of Pn and gs for SDI than RD carob trees; according to Tous et al. (2013), the cambium of the carob tree is active until September, hence, better physiological conditions in this period may result in a better overall status of the trees in the following productive year as well.

The study revealed that the RD system consistently exhibited higher gs values, particularly noticeable at specific stages of the vegetative cycle. This observation aligns with the findings of Ezzine et al. (2023), regarding the resilience of *C. siliqua* stomata under low water potentials, demonstrating their ability to maintain high relative water content through osmotic adjustment. Additionally, the notable Pn increase of the RD carob trees underscores their complex adaptive mechanisms for sustaining photosynthetic activity under drought conditions, a phenomenon previously documented by Lo Gullo and Salleo (1988). A similar trend was also observed in the later stage of the growth cycle, corroborating the patterns reported in Battle and Tous (1997).

## 5 Conclusions

This study successfully integrated machine learning, specifically the Random Forest model, with high-resolution satellite imagery to monitor carob trees’ physiological parameters (net assimilation, stomatal conductance and intrinsic water use efficiency). The approach presented in the study tries to provide technicians and farmers with a tool to reduce the time and labor typically required for field measurements, aligning with the need for efficient and representative data collection in agriculture. The significant role of specific spectral bands in predicting physiological parameters has been highlighted in the study. For instance, the yellow band was closely associated with Pn, highlighting its connection to photosynthetic rates and chlorophyll content, while the red band played a crucial role in predicting gs. The results of the research also indicated that carob trees might mitigate the stress caused by soil flooding through adaptive mechanisms. In addition, the importance of irrigation management in influencing Pn and gs, especially after harvest, has been demonstrated. The study’s findings contribute significantly to the understanding of carob tree-environment interactions and the potential of technology in enhancing agricultural productivity and resource management.

## Data availability statement

The raw data supporting the conclusions of this article will be made available by the authors, without undue reservation.

## Author contributions

SPG: Conceptualization, Data curation, Formal analysis, Investigation, Methodology, Software, Visualization, Writing – original draft, Writing – review & editing. VG: Methodology, Validation, Writing – review & editing. BL: Data curation, Investigation, Writing – review & editing. AG: Investigation, Writing – review & editing. GAV: Writing – review & editing. AT: Writing – review & editing. FPS: Funding acquisition, Project administration, Resources, Supervision, Validation, Writing – review & editing.
