# Supplementary material for: Predicting carob tree physiological parameters under different irrigation systems using Random Forest and Planet satellite images
Source: Front Plant Sci. 2024 Mar 19;15:1302435. doi: 10.3389/fpls.2024.1302435 (PMC10989058; doi:10.3389/fpls.2024.1302435)
Supplement: Supplementary file 1 [file DataSheet_1.pdf]

INFORME DE ENSAYO

Nº Muestra: 21064356

Nº Informe: 21064356.01

FITOSOIL Part of the Cotecna Group

PYME INNOVADORA

QS. Quality scheme for food.

ENAC ENSAYOS Nº 387/LE619 Nº 387/LE1330

Los ensayos marcados con (*), (***) y las opiniones, interpretaciones, etc... marcados con (**) no están amparados por la acreditación de ENAC.

Datos Cliente

CEBAS-CSIC

Campus Universitario, 25
30100 Espinardo Murcia (ESPAÑA)

Interlocutor: Francisco Pedrero

Ref.: **ALGARROBO 1**

Descripción: Suelo (2 kg aprox. en bolsa de plástico)

Matriz: Suelo

Datos Laboratorio

Descripción: Suelo (2 kg aprox. en bolsa de plástico)

Muestreo: Cliente

Recogida: Cliente - (Francisco Pedrero)

Condición:

Entrada: 30/06/2021 - 13:19 **Inicio:** 05/07/2021 **Finalización:** 12/07/2021

Obs.:

ANÁLISIS DE SUELO (físico-químico)

GRANULOMETRÍA (fracción <2mm)		Resultado		Textura (U.S.D.A)	Metodología
* Arena	(2-0,05 mm)	26	% (p/p)		Densímetro de Bouyoucos
* Limo	(0,05-0,002)	38	%(p/p)	**Franco arcilloso**	Densímetro de Bouyoucos
* Arcilla	(<0,002 mm)	36	%(p/p)		Densímetro de Bouyoucos
* Densidad aparente		1,331	g/cc		Cálculo matemático

SALINIDAD		Resultado		M.BAJO**	BAJO**	MEDIO**	ALTO**	M.ALTO**	
Conductividad elec.(25ºC) ext. acuoso 1/5 (p/v)		0,180	mS/cm						PTA-FQ-012, conductímetro, basado en UNE 77308
Cloruro sol. en extracto acuoso 1/5 (v/v)	Cl	< 0,070	meq/100g						PTA-FQ-012, c. iónica, basado en UNE-EN 10304-1
Sulfato sol. en extracto acuoso 1/5 (p/v)	Yeso	0,0055	%(p/p)						PTA-FQ-012, c. iónica, basado en UNE-EN 10304-1
Sodio asimilable	Na	0,140	meq/100g						PTA-FQ-009, BaCl2-TEA, ICP-AES, basado en ISO 22036
**REACCIÓN DEL SUELO**									
pH en KCl 1M extracto 1/2 (v/v)		7,67	Ud. pH						PTA-FQ-004, pH-metro, basado en UNE-EN 13027
* Caliza total	CaCO3	34,7	% CaCO3						PTA-FQ-154, analizador carbono inorgánico
* Caliza activa	CaCO3	10,75	% CaCO3						PTA-FQ-154, analizador carbono inorgánico
**MATERIA ORGÁNICA**									
* Materia orgánica total		2,47	%(p/p)						PTA-FQ-036, cálculo matemático
* Carbono orgánico total	C	1,43	%(p/p)						PTA-FQ-036, analizador elemental
* Relación carbono/nitrógeno total	C/N	7,4							Cálculo matemático, C.orgánico/N.total

Este informe sólo afecta a la muestra sometida a ensayo. En caso de que el laboratorio no sea el responsable del muestreo los resultados aplican a la muestra como se recibió. El cálculo de incertidumbres está a disposición del cliente. El laboratorio se hace responsable de las informaciones suministradas en este informe excepto las aportadas por el cliente y las opiniones y/o interpretaciones emitidas con carácter meramente informativo. Es responsabilidad del cliente la correcta interpretación de los resultados.
Este informe no deberá reproducirse total o parcialmente sin la aprobación por escrito de este laboratorio.

INFORME DE ENSAYO

Nº Muestra: 21064356

Nº Informe: 21064356.01

Los ensayos marcados con (*), (***) y las opiniones, interpretaciones, etc... marcados con (**) no están amparados por la acreditación de ENAC.

MACRONUTRIENTES PRIMARIOS		Resultado		M.BAJO**	BAJO**	MEDIO**	ALTO**	M.ALTO**	Metodología
Nitrógeno total	N	0,194	%(p/p)						PTA-FQ-036, Dumas, basado en UNE-EN 13654-2
Nitrógeno nítrico soluble ext. acuoso 1/5 (p/v)	N	12,8	mg/kg						PTA-FQ-012, c. iónica, basado en UNE-EN 10304-1
Nitrato soluble ext. acuoso 1/5 (p/v)	NO3	56,8	mg/kg						PTA-FQ-012. c. iónica, basado en UNE-EN 10304-1
Fósforo asimilable	P	11,8	mg/kg						PTA-FQ-015, Olsen, ICP-AES, basado en ISO 22036
Potasio asimilable	K	0,375	meq/100g						PTA-FQ-009, BaCl2-TEA, ICP-AES, basado en ISO 22036
**MACRONUTRIENTES SECUNDARIOS**									
Calcio asimilable	Ca	17,2	meq/100g						PTA-FQ-009, BaCl2-TEA, ICP-AES, basado en ISO 22036
Magnesio asimilable	Mg	0,91	meq/100g						PTA-FQ-009, BaCl2-TEA, ICP-AES, basado en ISO 22036
**MICRONUTRIENTES**									
Hierro asimilable	Fe	4,51	mg/Kg						PTA-FQ-010, ext. DPTA, ICP-AES, basado en ISO 22036
Manganeso asimilable	Mn	16,4	mg/Kg						PTA-FQ-010, ext. DPTA, ICP-AES, basado en ISO 22036
Zinc asimilable	Zn	0,79	mg/Kg						PTA-FQ-010, ext. DPTA, ICP-AES, basado en ISO 22036
Cobre asimilable	Cu	0,80	mg/Kg						PTA-FQ-010, ext. DPTA, ICP-AES, basado en ISO 22036
* Boro asimilable	B	0,243	mg/Kg						PTA-FQ-011,ext.acuosa, ICP-AES
**ESTUDIO DE LOS CATIONES ASIMILABLES**									
**Proporciones relativas**		**% Cat. asimilables**							
* Proporción relativa de sodio (PSI)		0,8							Cálculo matemático
* Proporción relativa de potasio		2,0							Cálculo matemático
* Proporción relativa de calcio		92,3							Cálculo matemático
* Proporción relativa de magnesio		4,9							Cálculo matemático
**Interacciones**		**Resultado**							
* Relación calcio/magnesio	Ca/Mg	18,9							Cálculo matemático
* Relación potasio/magnesio	K/Mg	0,41							Cálculo matemático

Este informe sólo afecta a la muestra sometida a ensayo. En caso de que el laboratorio no sea el responsable del muestreo los resultados aplican a la muestra como se recibió. El cálculo de incertidumbres está a disposición del cliente. El laboratorio se hace responsable de las informaciones suministradas en este informe excepto las aportadas por el cliente y las opiniones y/o interpretaciones emitidas con carácter meramente informativo. Es responsabilidad del cliente la correcta interpretación de los resultados.
Este informe no deberá reproducirse total o parcialmente sin la aprobación por escrito de este laboratorio.

FITOSOIL LABORATORIOS, S.L. - CIF: ESB 30553085 Inscrito en el Reg. Mercantil de Murcia, Tomo-1344, MU-23384, Folio 111. Colegiado por el COB con el Nº 6862-J
Pol.Ind.Oeste. c/ Alcalde Clemente García, parc.24/37. Mód.D-1 y D-2 - Envío Postal: Apdo. Correos 200 - 30169 - San Ginés-Murcia(España)
Tel.: +34 968 826809 · +34 968 883271/72 - Fax: +34 968 883278 - http://www.fitosoil.com - info@fitosoil.com

INFORME DE ENSAYO

Nº Muestra: 21064356

Nº Informe: 21064356.01

Los ensayos marcados con (*), (***) y las opiniones, interpretaciones, etc... marcados con (**) no están amparados por la acreditación de ENAC.

NUTRIENTES FERTILIZANTES (resumen)		Resultado				Metodología
Nitrógeno total	N	1.940	mg/kg	6.454	kg/Ha	PTA-FQ-036, Dumas, basado en UNE-EN 13654-2
Nitrógeno nítrico soluble ext. acuoso 1/5 (p/v)	N	12,8	mg/kg	42,7	kg/Ha	PTA-FQ-012. c. iónica, basado en UNE-EN 10304-1
Nitrato soluble ext. acuoso 1/5 (p/v)	NO3	56,8	mg/kg	189	kg/Ha	PTA-FQ-012, c. iónica, basado en UNE-EN 10304-1
Fósforo asimilable	P2O5	27,0	mg/kg	90	kg/Ha	PTA-FQ-015, Olsen, ICP-AES, basado en ISO 22036
Potasio asimilable	K2O	176	mg/kg	585	kg/Ha	PTA-FQ-009, BaCl2-TEA, ICP-AES, basado en ISO 22036
Calcio asimilable	CaO	4.819	mg/kg	16.034	kg/Ha	PTA-FQ-009, BaCl2-TEA, ICP-AES, basado en ISO 22036
Magnesio asimilable	MgO	184	mg/kg	611	kg/Ha	PTA-FQ-009, BaCl2-TEA, ICP-AES, basado en ISO 22036
Hierro asimilable	Fe	4,51	mg/kg	15,0	kg/Ha	PTA-FQ-010, ext. DPTA, ICP-AES, basado en ISO 22036
Manganeso asimilable	Mn	16,4	mg/kg	54	kg/Ha	PTA-FQ-010, ext. DPTA, ICP-AES, basado en ISO 22036
Zinc asimilable	Zn	0,79	mg/kg	2,64	kg/Ha	PTA-FQ-010, ext. DPTA, ICP-AES, basado en ISO 22036
Cobre asimilable	Cu	0,80	mg/kg	2,66	kg/Ha	PTA-FQ-010, ext. DPTA, ICP-AES, basado en ISO 22036
* Boro asimilable	B	0,243	mg/kg	0,81	kg/Ha	PTA-FQ-011,ext.acuosa, ICP-AES

Resultados obtenidos sobre muestra seca al aire y fracción <2mm. p/p: peso/peso. p/v: peso/volumen.

Ha: abreviatura referida a hectárea surco para una superficie de 10000 m^2 y una profundidad de 25 cm.

Los orientadores se establecen de modo general para un suelo con fines agronómicos, independientemente del tipo de cultivo y modalidad técnica empleada para la explotación del mismo.

Responsable Técnico Dpto. FÍSICO QUÍMICO
Bernardo Marín Romero

Director Técnico
Antonio Abellán Caravaca

FITOSOIL
Firmado digitalmente: Fitosoil Laboratorios S.L.
Fecha emisión: 12/07/2021 12:54:08

Este informe sólo afecta a la muestra sometida a ensayo. En caso de que el laboratorio no sea el responsable del muestreo los resultados aplican a la muestra como se recibió. El cálculo de incertidumbres está a disposición del cliente. El laboratorio se hace responsable de las informaciones suministradas en este informe excepto las aportadas por el cliente y las opiniones y/o interpretaciones emitidas con carácter meramente informativo. Es responsabilidad del cliente la correcta interpretación de los resultados.
Este informe no deberá reproducirse total o parcialmente sin la aprobación por escrito de este laboratorio.
