# Supplementary material for: Predicting carob tree physiological parameters under different irrigation systems using Random Forest and Planet satellite images
Source: Front Plant Sci. 2024 Mar 19;15:1302435. doi: 10.3389/fpls.2024.1302435 (PMC10989058; doi:10.3389/fpls.2024.1302435)
Supplement: Supplementary file 2 [file DataSheet_2.pdf]

INFORME DE ENSAYO

Nº Muestra: 21064357

Nº Informe: 21064357.01

Los ensayos marcados con (*), (***) y las opiniones, interpretaciones, etc... marcados con (**) no están amparados por la acreditación de ENAC.

Datos Cliente

CEBAS-CSIC

Campus Universitario, 25
30100 Espinardo Murcia (ESPAÑA)

Interlocutor: Francisco Pedrero

Ref.: **ALGARROBO 2**

Descripción: Suelo (2 kg aprox. en bolsa de plástico)

Matriz: Suelo

Datos Laboratorio

Descripción: Suelo (2 kg aprox. en bolsa de plástico)

Muestreo: Cliente

Recogida: Cliente - (Francisco Pedrero)

Condición:

Entrada: 30/06/2021 - 13:19 **Inicio:** 05/07/2021 **Finalización:** 12/07/2021

Obs.:

ANÁLISIS DE SUELO (físico-químico)

GRANULOMETRÍA (fracción <2mm)		Resultado		Textura (U.S.D.A)	Metodología
* Arena	(2-0,05 mm)	48	% (p/p)		Densímetro de Bouyoucos
* Limo	(0,05-0,002)	30	%(p/p)	Franco	Densímetro de Bouyoucos
* Arcilla	(<0,002 mm)	22	%(p/p)		Densímetro de Bouyoucos
* Densidad aparente		1,491	g/cc		Cálculo matemático

SALINIDAD		Resultado		M.BAJO**	BAJO**	MEDIO**	ALTO**	M.ALTO**	
Conductividad elec.(25ºC) ext. acuoso 1/5 (p/v)		0,154	mS/cm						PTA-FQ-012, conductímetro, basado en UNE 77308
Cloruro sol. en extracto acuoso 1/5 (v/v)	Cl	< 0,070	meq/100g						PTA-FQ-012, c. iónica, basado en UNE-EN 10304-1
Sulfato sol. en extracto acuoso 1/5 (p/v)	Yeso	< 0,00448	%(p/p)						PTA-FQ-012, c. iónica, basado en UNE-EN 10304-1
Sodio asimilable	Na	< 0,087	meq/100g						PTA-FQ-009, BaCl2-TEA, ICP-AES, basado en ISO 22036
**REACCIÓN DEL SUELO**									
pH en KCl 1M extracto 1/2 (v/v)		7,85	Ud. pH						PTA-FQ-004, pH-metro, basado en UNE-EN 13027
* Caliza total	CaCO3	40,4	% CaCO3						PTA-FQ-154, analizador carbono inorgánico
* Caliza activa	CaCO3	6,59	% CaCO3						PTA-FQ-154, analizador carbono inorgánico
**MATERIA ORGÁNICA**									
* Materia orgánica total		1,108	%(p/p)						PTA-FQ-036, cálculo matemático
* Carbono orgánico total	C	0,643	%(p/p)						PTA-FQ-036, analizador elemental
* Relación carbono/nitrógeno total	C/N	5,4							Cálculo matemático, C.orgánico/N.total

Este informe sólo afecta a la muestra sometida a ensayo. En caso de que el laboratorio no sea el responsable del muestreo los resultados aplican a la muestra como se recibió. El cálculo de incertidumbres está a disposición del cliente. El laboratorio se hace responsable de las informaciones suministradas en este informe excepto las aportadas por el cliente y las opiniones y/o interpretaciones emitidas con carácter meramente informativo. Es responsabilidad del cliente la correcta interpretación de los resultados.
Este informe no deberá reproducirse total o parcialmente sin la aprobación por escrito de este laboratorio.

Los ensayos marcados con (*), (***) y las opiniones, interpretaciones, etc... marcados con (**) no están amparados por la acreditación de ENAC.

MACRONUTRIENTES PRIMARIOS		Resultado		M.BAJO**	BAJO**	MEDIO**	ALTO**	M.ALTO**	Metodología
Nitrógeno total	N	0,119	%(p/p)						PTA-FQ-036, Dumas, basado en UNE-EN 13654-2
Nitrógeno nítrico soluble ext. acuoso 1/5 (p/v)	N	9,2	mg/kg						PTA-FQ-012, c. iónica, basado en UNE-EN 10304-1
Nitrato soluble ext. acuoso 1/5 (p/v)	NO3	40,7	mg/kg						PTA-FQ-012. c. iónica, basado en UNE-EN 10304-1
Fósforo asimilable	P	< 10,0	mg/kg						PTA-FQ-015, Olsen, ICP-AES, basado en ISO 22036
Potasio asimilable	K	0,350	meq/100g						PTA-FQ-009, BaCl2-TEA, ICP-AES, basado en ISO 22036
**MACRONUTRIENTES SECUNDARIOS**									
Calcio asimilable	Ca	10,6	meq/100g						PTA-FQ-009, BaCl2-TEA, ICP-AES, basado en ISO 22036
Magnesio asimilable	Mg	0,55	meq/100g						PTA-FQ-009, BaCl2-TEA, ICP-AES, basado en ISO 22036
**MICRONUTRIENTES**									
Hierro asimilable	Fe	3,07	mg/Kg						PTA-FQ-010, ext. DPTA, ICP-AES, basado en ISO 22036
Manganeso asimilable	Mn	13,4	mg/Kg						PTA-FQ-010, ext. DPTA, ICP-AES, basado en ISO 22036
Zinc asimilable	Zn	0,73	mg/Kg						PTA-FQ-010, ext. DPTA, ICP-AES, basado en ISO 22036
Cobre asimilable	Cu	0,67	mg/Kg						PTA-FQ-010, ext. DPTA, ICP-AES, basado en ISO 22036
* Boro asimilable	B	0,175	mg/Kg						PTA-FQ-011,ext.acuosa, ICP-AES
**ESTUDIO DE LOS CATIONES ASIMILABLES**									
**Proporciones relativas**		**% Cat. asimilables**							
* Proporción relativa de sodio (PSI)		0,4							Cálculo matemático
* Proporción relativa de potasio		3,0							Cálculo matemático
* Proporción relativa de calcio		91,8							Cálculo matemático
* Proporción relativa de magnesio		4,8							Cálculo matemático
**Interacciones**		**Resultado**							
* Relación calcio/magnesio	Ca/Mg	19,2							Cálculo matemático
* Relación potasio/magnesio	K/Mg	0,63							Cálculo matemático

Este informe sólo afecta a la muestra sometida a ensayo. En caso de que el laboratorio no sea el responsable del muestreo los resultados aplican a la muestra como se recibió. El cálculo de incertidumbres está a disposición del cliente. El laboratorio se hace responsable de las informaciones suministradas en este informe excepto las aportadas por el cliente y las opiniones y/o interpretaciones emitidas con carácter meramente informativo. Es responsabilidad del cliente la correcta interpretación de los resultados.
Este informe no deberá reproducirse total o parcialmente sin la aprobación por escrito de este laboratorio.

Los ensayos marcados con (*), (***) y las opiniones, interpretaciones, etc... marcados con (**) no están amparados por la acreditación de ENAC.

NUTRIENTES FERTILIZANTES (resumen)		Resultado				Metodología
Nitrógeno total	N	1.190	mg/kg	4.435	kg/Ha	PTA-FQ-036, Dumas, basado en UNE-EN 13654-2
Nitrógeno nítrico soluble ext. acuoso 1/5 (p/v)	N	9,2	mg/kg	34,3	kg/Ha	PTA-FQ-012. c. iónica, basado en UNE-EN 10304-1
Nitrato soluble ext. acuoso 1/5 (p/v)	NO3	40,7	mg/kg	152	kg/Ha	PTA-FQ-012, c. iónica, basado en UNE-EN 10304-1
Fósforo asimilable	P2O5	< 22,9	mg/kg	< 85	kg/Ha	PTA-FQ-015, Olsen, ICP-AES, basado en ISO 22036
Potasio asimilable	K2O	164	mg/kg	612	kg/Ha	PTA-FQ-009, BaCl2-TEA, ICP-AES, basado en ISO 22036
Calcio asimilable	CaO	2.969	mg/kg	11.065	kg/Ha	PTA-FQ-009, BaCl2-TEA, ICP-AES, basado en ISO 22036
Magnesio asimilable	MgO	111	mg/kg	414	kg/Ha	PTA-FQ-009, BaCl2-TEA, ICP-AES, basado en ISO 22036
Hierro asimilable	Fe	3,07	mg/kg	11,5	kg/Ha	PTA-FQ-010, ext. DPTA, ICP-AES, basado en ISO 22036
Manganeso asimilable	Mn	13,4	mg/kg	50	kg/Ha	PTA-FQ-010, ext. DPTA, ICP-AES, basado en ISO 22036
Zinc asimilable	Zn	0,73	mg/kg	2,71	kg/Ha	PTA-FQ-010, ext. DPTA, ICP-AES, basado en ISO 22036
Cobre asimilable	Cu	0,67	mg/kg	2,48	kg/Ha	PTA-FQ-010, ext. DPTA, ICP-AES, basado en ISO 22036
* Boro asimilable	B	0,175	mg/kg	0,65	kg/Ha	PTA-FQ-011,ext.acuosa, ICP-AES

Resultados obtenidos sobre muestra seca al aire y fracción <2mm. p/p: peso/peso. p/v: peso/volumen.

Ha: abreviatura referida a hectárea surco para una superficie de 10000 m^2 y una profundidad de 25 cm.

Los orientadores se establecen de modo general para un suelo con fines agronómicos, independientemente del tipo de cultivo y modalidad técnica empleada para la explotación del mismo.

Responsable Técnico Dpto. FÍSICO QUÍMICO
Bernardo Marín Romero

Director Técnico
Antonio Abellán Caravaca

FITOSOIL Firmado digitalmente: Fitosoil Laboratorios S.L. Fecha emisión: 12/07/2021 12:54:09
